# Multi-criteria decision support framework for sustainable implementation of effective green supply chain management practices

**DOI:** 10.1186/s40064-016-2233-2

**Published:** 2016-05-20

**Authors:** Omar Boutkhoum, Mohamed Hanine, Hicham Boukhriss, Tarik Agouti, Abdessadek Tikniouine

**Affiliations:** Department of Computer Science, Faculty of the Sciences Semlalia, Cadi Ayyad University, Marrakech, Morocco; MTI Lab. ENSA School, Cadi Ayyad University, Safi, Morocco

**Keywords:** Green supply chain management (GSCM) practices, FAHP, Fuzzy TOPSIS, Multi-criteria decision-making, Decision support system

## Abstract

At present, environmental issues become real critical barriers for many supply chain corporations concerning the sustainability of their businesses. In this context, several studies have been proposed from both academia and industry trying to develop new measurements related to green supply chain management (GSCM) practices to overcome these barriers, which will help create new environmental strategies, implementing those practices in their manufacturing processes. The objective of this study is to present the technical and analytical contribution that multi-criteria decision making analysis (MCDA) can bring to environmental decision making problems, and especially to GSCM field. For this reason, a multi-criteria decision-making methodology, combining fuzzy analytical hierarchy process and fuzzy technique for order preference by similarity to ideal solution (fuzzy TOPSIS), is proposed to contribute to a better understanding of new sustainable strategies through the identification and evaluation of the most appropriate GSCM practices to be adopted by industrial organizations. The fuzzy AHP process is used to construct hierarchies of the influential criteria, and then identify the importance weights of the selected criteria, while the fuzzy TOPSIS process employs these weighted criteria as inputs to evaluate and measure the performance of each alternative. To illustrate the effectiveness and performance of our MCDA approach, we have applied it to a chemical industry corporation located in Safi, Morocco.

## Background

The risks of supply chain management (SCM) are increasingly spotlighted in many studies due to the critical events of many environmental issues (Chen et al. 2014; Wagner and Kemmerling 2014; Subramanian and Gunasekaran 2015; Dadhich et al. 2015). In fact, the industry practitioners and policy makers are under increasing pressure to continuously reduce the negative environmental impact of their supply chains. In this regard, several corporations, researchers and practitioners in industry have attempted to find out variables that influence either positively or negatively SCM, with a view to make businesses more sustainable environmentally. In the same context, customers are also becoming more and more concerned about the environment and are making procurement choices with an eye on environmentally friendliness, taking as examples, environmental logistics (short distance goods delivery), choices of environmentally friendly packaging, and recourse to ecological logistics modes (Jayaram and Avittathur 2015).

For these reasons, the green supply chain management (GSCM), as a field of ecological economics (sustainability), is recognized as a direct and effective mechanism to address environmental problems. It represents the combination of the supply chain management and environmental thinking encompassing material sourcing and selection, product design, manufacturing processes and delivery of the final product to the consumer (Srivastava 2007). Compared to SCM, grouping all activities related to manufacturing from raw material acquisition until delivery of the final product, GSCM aims to control and minimize waste in the industrial system so as to save energy and prevent the dissipation of harmful substances into the environment. In addition, GSCM is an integration of natural environmental concerns into SCM through the implementation of diverse green solutions and practices like green design, life cycle analysis, green purchasing, green logistics, environmental technologies, and collaborative practices with suppliers, distributors, and customers (Jayant and Azhar 2014; Govindan et al. 2015a, b). GSCM has emerged as an important organizational philosophy that tries to reduce environmental impacts and risks (Hu and Hsu 2010; Azevedo et al. 2011). Indeed, more efforts have been dedicated to the assessment of the GSCM ability with respect to its impact on the environmental performance of a given corporation as well as supporting its competitive strategies (Testa and Iraldo 2010). Other studies have investigated the effect of GSCM, for example, on geographical areas (Zhu and Sarkis 2004) and industrial branches (Zhu and Sarkis 2007; Shang et al. 2010). Lee et al. (2014a, b) propose to create technological innovation through GSCM practices, and they conclude that internal environmental management and eco-design are significantly and positively related to technological innovation. Evidently, internal GSCM practices and external green collaboration have significant impacts on green performance, which leads to improve firm competitiveness (Yang et al. 2013).

Consequently, the continued academic growth of this inceptive field and its development requires that new insights and knowledge be generated. Hence, many GSCM practices have been proposed taking into account all information and knowledge generated during the manufacturing process and among all supplier partners. In fact, organizations should become greener (Marcus and Fremeth 2009) by implementing many GSCM practices which consist in several environmental supply chain management directives that can be adopted both inside and outside the company (Ageron et al. 2012). This leads to reach a win–win perspective (Hart and Dowell 2011) by enabling firms to create and generate more business opportunities (Wang and Chan 2013).

However, applying these practices and selecting the most appropriate ones for implementation is becoming increasingly difficult due to many barriers (Jayant and Azhar 2014; Govindan et al. 2014), seeing that each organization has its own strategies, purposes and capabilities to consider. In addition, lack of information availability leads organizations to make decisions under significant uncertainty causing unexpected results. As such, dealing with uncertain and heterogeneous information requires a systematic framework to collect and organize technical and analytical information. Hence, the multi-criteria decision-making (MCDA) methods combined with fuzzy set theory are involved to provide a systematic methodology for adequate evaluation of sustainable supply chain management practices.

MCDA allows decision makers to face complex decision making situations involving multiple, usually conflicting decision criteria which include quantitative and/or qualitative aspects in a decision-making process. Many MCDA methods are available to decision makers and analysts aiming to support decision making in the GSCM practices. These methods can be grouped into two approaches: methods of the unique approach of synthesis such as TOPSIS, SMART, Weighted sum, MAUT, MAVT, UTA, AHP, ANP and the outranking methods of synthesis as PROMETHEE, ELECTRE and ORESTE etc. (Boutkhoum et al. 2015a). In this context, the use of a decision-making methodology based on fuzzy analytical hierarchy process (FAHP) and fuzzy technique for order preference by similarity to ideal solution (fuzzy TOPSIS), to firstly identify the best GSCM practices, and then evaluate and rank these practices according to their importance and their impact on the strategic evolution of organizations adopting them, has not received much interest in terms of scientific research, and particularly for its application in the Moroccan regions. In fact, the leap from supply chain management to green supply chain management has been relatively rare in the Moroccan context. These reasons have motivated us to propose and develop the multi-criteria decision support framework based on FAHP and fuzzy TOPSIS to identify, evaluate and rank the ideal GSCM practices. During the assessment process of the GSCM practices, ten criteria and ten alternatives have been considered. The identification of these criteria and alternatives is performed on the basis of the literature review, brainstorming and discussion between three decision group members. Based on the judgments of the decision group members, FAHP process is used to determine the importance weight and rank the evaluation criteria, when fuzzy TOPSIS process is employed to evaluate and measure the performance of each GSCM practice. The proposed framework is also adapted to model the imprecision and linguistic vagueness when making decisions. An empirical case study of a chemical industry corporation located in Safi, Morocco, will be presented in order to illustrate the effectiveness of our proposed approach which is implemented by using Microsoft Excel as software.

The remainder of this paper is organized as follows. In “Literature review” section, literature work is presented. “Research methodology” section discusses our research methodology and develops our proposed approach. An empirical study illustrating the effectiveness and performance of our decisional framework is presented later in “Numerical illustration” section. Finally “Conclusion” section contains some concluding remarks.

## Literature review

In this section, we briefly highlight some environmental applications of MCDA, particularly in the field of GSCM. The majority of those applications have treated problems, success factors, performance measurements and barriers related to the implementation and development of GSCM as a sustainable research area. According to Huang et al. (2011), the various application of MCDA in the environmental field from 1990 to 2010 has experienced significant evolutions. Also, in terms of the total number of papers published, AHP/ANP dominates MCDA methods followed by MAUT, ELECTRE and PROMETHEE, respectively. The majority of MCDA methods are incorporated with fuzzy set theory (FAHP, Fuzzy TOPSIS, Fuzzy PROMETHEE…) to combine human heuristics into computer-assisted decision making, and represent the human thinking and interpretation by establishing mathematical rules for working with numerical data and linguistic terms, which are easier to understand for human reasoning. Those methods are also uniformly distributed among several application areas. Concerning GSCM application areas, we quote for example, the contribution of Govindan et al. (2015a, b) which propose an intuitionistic fuzzy based DEMATEL (DEcision-MAking Trial and Evaluation Laboratory) method for developing green performances and practices in a GSCM. The authors have evaluated GSCM practices using DEMATEL method to find the main practices to enhance both economic and environmental performances. Mathiyazhagan et al. (2013), propose an interpretive structural modeling approach for the barrier analysis by removing the dominant barrier that acts on the adoption of green concept in the supply chain of a company. In a parallel way, an analysis model of the drivers affecting the implementation of GSCM is presented by Diabata and Govindan (2011). The contributions of Luthra et al. (2014) and Rozar et al. (2015) have focused on selecting the success factors of GSCM for a better achievement of sustainability with different illustrations of case studies.

Moreover, the performance measurement for GSCM is introduced by Hervani et al. (2005), while the ranking of those performance measures towards sustainability is provided by Garg et al. (2014), using AHP method. Fuzzy AHP and TOPSIS methodologies have been widely used in many contributions, as example, Muralidhar et al. (2012) for the evaluation of GSCM strategies, and then by Patil and Kant (2014) for the ranking of the solutions of knowledge management adoption in supply chain to overcome its barriers. The selection of green suppliers for a Brazilian electronics company is built on the criteria of GSCM practices using fuzzy TOPSIS methodology (Kannan et al. 2014). Furthermore, Yeh and Chuang (2011) have developed an optimum mathematical planning model for partner selection in green supply chain problems using multi-objective genetic algorithm. In the same context, a detailed procedure to solve complex problems of GSCM strategy-selection and evaluate the most appropriate activity in each business function using analytic network process (ANP) is presented by Chen et al. (2012). Several other researches such as Eshtehardian et al. (2013), Kepaptsoglou et al. (2013) and Kim et al. (2014) have combined AHP and/or ANP with fuzzy set theory in their studies, especially for hierarchically structuring the problem and assigning weight to criteria by taking into consideration human thoughts when making the best decision.

However, although corporations consider environmental management their strategic priority, evaluating and measuring the performance of GSCM in terms of implemented practices has attracted little attention especially by using multi-criteria decision framework based on fuzzy AHP-TOPSIS methods. Hence, we propose in this paper a multi-criteria decision support framework for sustainable implementation of effective green supply chain management practices. The followed methodology and its application are briefly described in the following section.

## Research methodology

Before processing the principle of FAHP and fuzzy TOPSIS, as a powerful decision-making methodology, we briefly review the concept of fuzzy theory as defined by Zadeh (1965).

### Fuzzy set and fuzzy numbers

#### **Definition 1**

 A fuzzy set A of a universe of discourse X is characterized by a membership function:

If μ_A_ is the membership function of the fuzzy set A, ∀x ∈ X μ_A_ ∈ [0, 1].

The set A is defined by A = {(x, μ_A_ (x))|x ∈ X}.

#### **Definition 2**

**Fig. 1:**
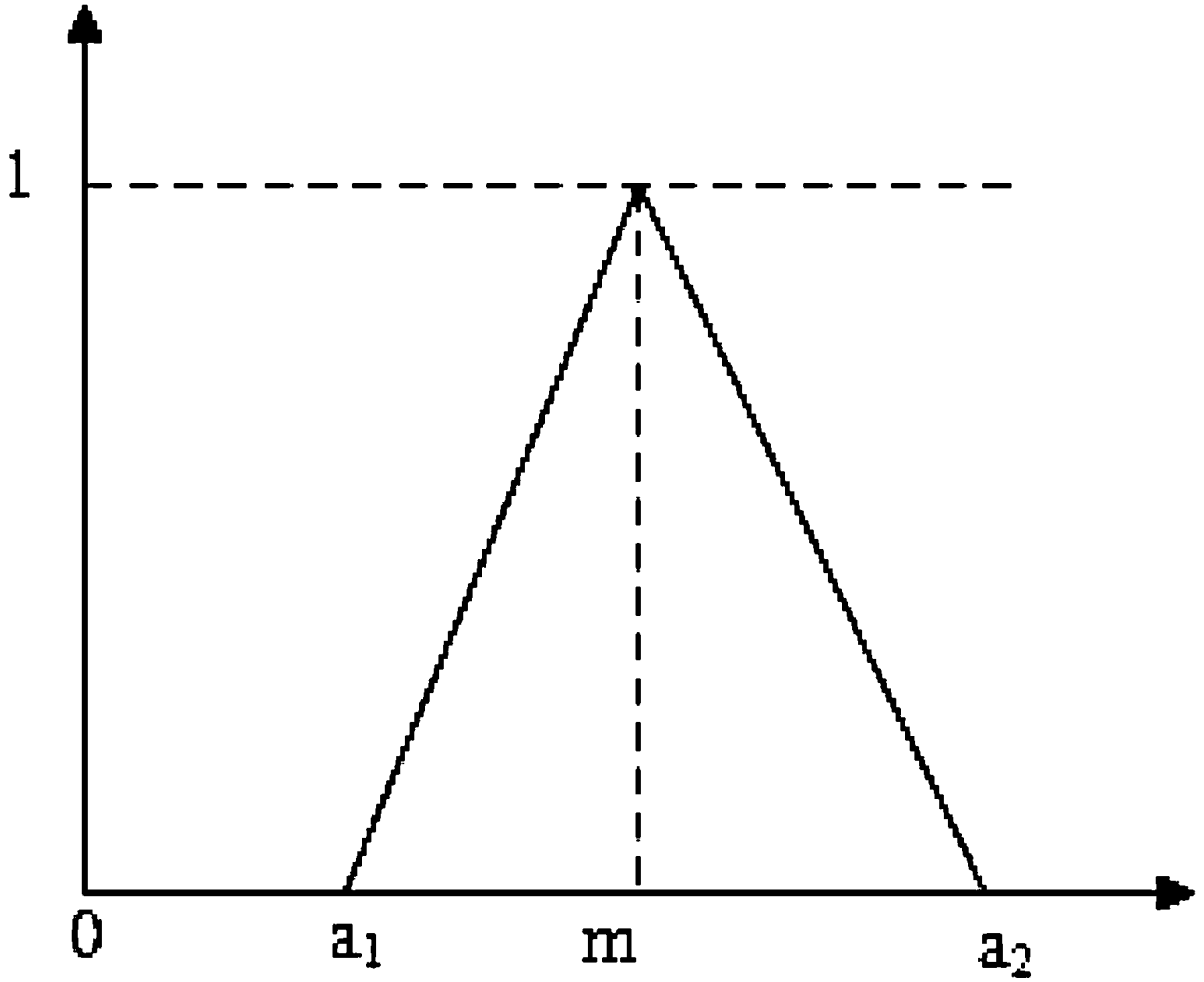
Triangular fuzzy number A = (a_1_, m, a_2_)

 Triangular Fuzzy Numbers (TFNs) is among the most popular shapes of fuzzy number. It is represented with triplet (a_1_, m, a_2_) such that a_1_ ≤ m ≤ a_2_ as shown in Fig. 1. Its mathematical form is shown by Eq. (1).

1 $$\upmu_{\text{M}} \left( x \right) = \left\{ \begin{aligned} 0, \quad x \le a_{1} \hfill \\ (x - a_{1} )/(m - a_{1} ), \quad a_{1} < x \le m \hfill \\ (a_{2} - x)/(a_{2} - m), \quad m < x \le a_{2} \hfill \\ 0, \quad x > a_{2} \hfill \\ \end{aligned} \right.$$

The basic operations on fuzzy triangular numbers are as follows:

2 $$\left( {a_{1} ,m_{1} ,a_{2} } \right) + \left( {b_{1} ,m_{2} ,b_{2} } \right) = \left( {a_{1} + b_{1} ,m_{1} + m_{2} ,a_{2} + b_{2} } \right)$$

3 $$\left( {a_{1} ,m_{1} ,a_{2} } \right)*\left( {b_{1} ,m_{2} ,b_{2} } \right) = \left( {a_{1} *b_{1} ,m_{1} *m_{2} ,a_{2} *b_{2} } \right)$$

4 $$\left( {a_{1} ,m_{1} ,a_{2} } \right)/\left( {b_{1} ,m_{2} ,b_{2} } \right) = \left( {a_{1} /b_{2} ,m_{1} /m_{2} ,a_{2} /b_{1} } \right)$$

5 $$\left( {a_{1} ,m_{1} ,a_{2} } \right)^{ - 1} = \left( {1/a_{2} ,1/m_{1} ,1/a_{1} } \right)$$

6 $$\left( {a_{1} ,m_{1} ,a_{2} } \right)^{1/n} = \left( {a_{1}^{1/n} ,m_{1}^{1/n} ,a_{2}^{1/n} } \right)$$

For *a*_1_, *a*_2_ > 0; *m*_1_, *m*_2_ > 0; *b*_1_, *b*_2_ > 0.

### The principle of the FAHP method

The analytic hierarchy process (AHP) introduced by Saaty (1980) is a multi-criteria decision-making method that structures hierarchically a multi period and multi-criteria problem so that solutions are facilitated. This method is based on an approach representing a decision problem by a hierarchical structure reflecting the interactions between the various elements of the problem. However, the application of Saaty’s AHP has some shortcomings (Yang and Chen 2004; Boutkhoum et al. 2015b) as follows: (1) the AHP method deals with the very unbalanced scale of judgment, (2) the ineffectiveness of AHP when applied to an ambiguous problem, (3) the AHP method’s ranking is rather imprecise and the use of the discrete scale of AHP is easy and simple but does not take into account the uncertainty associated with the mapping of human judgment to a number by natural language. As a result, several researchers starting by Buckley (1985) incorporated the fuzzy set theory into the traditional AHP. Thus, FAHP has become a suitable tool to address the uncertainty of human cognition and judgment, ambiguity knowledge and multiple criteria in real-world multi-criteria decision-making problems (Kabir and Sumi 2014; Kilic et al. 2014; Somsuk and Laosirihongthong 2014; Chen et al. 2015). In the present study, we propose to utilize the FAHP methodology that determines the importance weights of selected criteria.

The various computational steps involved in employing the FAHP methodology (Buckley 1985; Gil-Lafuente et al. 2014) are described as below:

*Step 1* the problem must be decomposed into a hierarchy of interrelated elements (factors and sub-factors). At the top of the hierarchy we find the goal, the elements contributing to achieve it are in the lower levels.

*Step 2* The comparison matrix *D* is built by conducting pairwise comparisons of the elements of each hierarchical level with respect to an element of the upper hierarchical level.

7 $$D = \left[ {{\text{x}}_{ij} } \right] = \left[ {\begin{array}{*{20}r} \hfill {x_{11} } & \quad \hfill {x_{12} } & \quad \hfill {x_{13} } & \quad \hfill \cdots & \quad \hfill {x_{1n} } \\ \hfill {x_{21} } & \quad \hfill {x_{22} } & \quad \hfill {x_{23} } & \quad \hfill \cdots & \quad \hfill {x_{2n} } \\ \hfill {x_{31} } & \quad \hfill {x_{32} } & \quad \hfill {x_{33} } & \quad \hfill \cdots & \quad \hfill {x_{3n} } \\ \hfill \vdots & \quad \hfill \vdots & \quad \hfill \vdots & \quad \hfill \vdots & \quad \hfill \vdots \\ \hfill {x_{n1} } & \quad \hfill {x_{n2} } & \quad \hfill {x_{n3} } & \quad \hfill \cdots & \quad \hfill {x_{nn} } \\ \end{array} } \right]$$

where n = criteria number to be evaluated, x_ij_ = importance of ith criteria according to jth criteria.

*Step 3* The triangular fuzzy numbers (TFNs) must be established using the geometric average to represent the consensus of most decision group members. They were established by integrating fuzzy opinions on the relative importance of paired elements. The reason for using TFNs to capture the vagueness of the linguistic assessments is that TFN is intuitively easy to use (Tsao and Chu 2001; Kannan et al. 2009).

$$\tilde{x}_{ij} = (a_{1ij} ,m_{ij} ,a_{2ij} ),\quad a_{1ij} \le m_{ij} \le a_{2ij} ,\quad {\text{i}},{\text{ j}} = 1,2 \ldots ,{\text{n}}$$

8 $$a_{1ij} = Min(a_{1ijk} )\quad {\text{k}} = 1,2, \ldots ,{\text{n}}$$

9 $$m_{ij} = \left( {\prod\limits_{k = 1}^{n} {{\text{m}}_{ijk} } } \right)^{1/n}$$

10 $$a_{2ij} = Max(a_{2ijk} )\quad {\text{k}} = 1,2, \ldots ,{\text{n}}$$

where $(a_{1ijk} ,m_{ijk} ,a_{2ijk} )$ is the fuzzy evaluation of member K on the relative importance of criteria i and j.

*Step 4* After establishing triangular fuzzy numbers to evaluate experts’ fuzzy opinions, a fuzzy positive reciprocal matrix $\tilde{D}$ must be established as follows:

11 $$\tilde{D} = \left[ {{\tilde{\text{x}}}_{ij} } \right] = \left[ {\begin{array}{*{20}r} \hfill {\tilde{x}_{11} } & \quad \hfill {\tilde{x}_{12} } & \quad \hfill {\tilde{x}_{13} } & \quad \hfill \cdots & \quad \hfill {\tilde{x}_{1n} } \\ \hfill {\tilde{x}_{21} } & \quad \hfill {\tilde{x}_{22} } & \quad \hfill {\tilde{x}_{23} } & \quad \hfill \cdots & \quad \hfill {\tilde{x}_{2n} } \\ \hfill {\tilde{x}_{31} } & \quad \hfill {\tilde{x}_{32} } & \quad \hfill {\tilde{x}_{33} } & \quad \hfill \cdots & \quad \hfill {\tilde{x}_{3n} } \\ \hfill \vdots & \quad \hfill \vdots & \quad \hfill \vdots & \quad \hfill \vdots & \quad \hfill \vdots \\ \hfill {\tilde{x}_{n1} } & \quad \hfill {\tilde{x}_{n2} } & \quad \hfill {\tilde{x}_{n3} } & \quad \hfill \cdots & \quad \hfill {\tilde{x}_{nn} } \\ \end{array} } \right],\quad {\text{i}},{\text{j}} = 1,2, \ldots ,{\text{n}}$$

*Step 5* For the consistency verification of fuzzy matrix $\tilde{D}$:

We assume that $D = \left[ {x_{ij} } \right]$ is a positive reciprocal matrix and its corresponding fuzzy positive reciprocal matrix is $\tilde{D} = \left[ {\tilde{x}_{ij} } \right]$. Therefore, $D = \left[ {x_{ij} } \right]$ is consistent, as well as $\tilde{D} = \left[ {\tilde{x}_{ij} } \right].$

*Step 6* The fuzzy weight $\left( {\tilde{W}_{i} } \right)$ of the fuzzy positive reciprocal matrix is calculated as explained below:

12 $$\tilde{Z}_{i} = \left[ {\prod\nolimits_{j = 1}^{n} {\tilde{x}_{ij} } } \right]^{1/n} ,\quad {\text{i}},{\text{j}} = 1,2, \ldots ,{\text{n}}$$

13 $$\tilde{W}_{i} = \tilde{Z}_{i} \otimes \left( {\sum\limits_{i = 1}^{n} {\tilde{Z}_{i} } } \right)^{ - 1}$$

$\tilde{Z}_{i}$: geometric average of triangle fuzzy numbers.

*Step 7* During this step, we conduct a defuzzification process using the gravity method as follows:

14 $$W_{i} = \frac{{W_{{a_{1} i}} \oplus W_{mi} \oplus W_{{a_{2} i}} }}{3}$$

$W_{{a_{1} i}}$: the value of the minimum fuzzy weight (left value). $W_{mi}$: the value of the grade of membership of the fuzzy weight. $W_{{a_{2} i}}$: the value of the maximum fuzzy weight (right value). $W_{i}$: convert the fuzzy weight of the triangular fuzzy numbers into a single value.

*Step 8* The final normalized weight (NW) is then obtained as follows:

15 $$NW_{i} = \frac{{W_{i} }}{{\sum\nolimits_{i = 1}^{n} {W_{i} } }}$$

### Fuzzy TOPSIS

TOPSIS approach is one of the classical multi-criteria decision-making methods developed by Hwang and Yoon (1981) for ranking problems in real time situations. In fact, the chosen alternative should have the shortest distance from the positive ideal solution and the farthest distance from the negative ideal solution. Different researchers have successfully used the TOPSIS method to analyze different multi criteria problems (Nilashi and Ibrahim 2013; Bilbao-Terol et al. 2014; Tavana et al. 2015; Hanine et al. 2016). The main limitation of the TOPSIS method consists in the inability to capture the ambiguity or inaccuracy inherent in a group decision making situation (Chen 2000). To overcome this shortcoming, fuzzy set theory incorporated with the traditional TOPSIS method may be a better approach allowing decision makers to integrate incomplete and unquantifiable information. Fuzzy TOPSIS is a suitable tool for solving group decision making situations under the fuzzy environment (Chen 2000; Patil and Kant 2014; Onar et al. 2014). It attempts to integrate vital qualitative attributes in performance analysis of GSCM practices and transforms qualitative data into equivalent quantitative measures. A brief review of fuzzy TOPSIS method used and applied in environmental application fields is presented in Table 1.

**Table 1:** A review of fuzzy TOPSIS method used and applied in environmental application fields

Researcher (year)	Modeling techniques used	Issues addressed
Büyüközkan and Çifçi (2012)	Fuzzy DEMATEL, fuzzy ANP and fuzzy TOPSIS	Evaluate green suppliers
Shen et al. (2013)	Fuzzy TOPSIS	Evaluating green supplier’s performance in GSC
Bas (2013)	SWOT-fuzzy TOPSIS methodology combined with AHP	Analysis of electricity supply chain
Govindan et al. (2013)	Fuzzy TOPSIS	Measuring sustainability performance of a supplier
Taylan et al. (2014)	Fuzzy AHP and fuzzy TOPSIS	Construction projects selection and risk assessment
Mangla et al. (2015)	Fuzzy AHP and fuzzy TOPSIS	Prioritize the responses of risks in GSC
Tyagi et al. (2015)	Fuzzy TOPSIS	Improve Performance of GSCM System
Kusi-Sarpong et al. (2015)	Rough set theory elements and fuzzy TOPSIS	GSC practices evaluation in the mining industry
Lima-Junior and Carpinetti (2016)	SCOR metrics and fuzzy TOPSIS	Aid supplier evaluation and management
Wood (2016)	Fuzzy and intuitionistic fuzzy TOPSIS	Supplier selection

The algorithm of fuzzy TOPSIS (modified from Tyagi et al. 2015; Lima-Junior and Carpinetti 2016) can be summarized as in the following steps:

*Step 1* This step concerns the weights of evaluation criteria which are already determined using FAHP method.

*Step 2* Establish a fuzzy decision matrix to rate ‘m’ alternatives with respect to each criterion (‘n’ criteria) as given below:16where g_1_, g_2_, …, g_m_ = feasible alternatives, C_1_, C_2_, …, C_n_ = evaluation criteria, $\tilde{g}_{ij}$ = the rating given to alternative g_i_ against criterion C_j_.

*Step 3* Construct the normalized fuzzy decision matrix $\tilde{r}_{ij}$ as follows:

17 $$\left. {\begin{array}{ll} & \tilde{r}_{ij} = \left( {\frac{{a_{1ij} }}{{a_{2j}^{ + } }},\frac{{m_{ij} }}{{a_{2j}^{ + } }},\frac{{a_{2ij} }}{{a_{2j}^{ + } }}} \right), \\ & a_{2j}^{ + } = \mathop {\hbox{max} }\limits_{i} a_{2ij} \,\left( {\text{benefit criteria}} \right)\\ \end{array}} \right\}$$

18 $$\left. {\begin{array}{ll} & \tilde{r}_{ij} = \left( {\frac{{a_{1j}^{ - } }}{{a_{2ij} }},\frac{{a_{1j}^{ - } }}{{m_{ij} }},\frac{{a_{1j}^{ - } }}{{a_{1ij} }}} \right), \hfill \\ &a_{1j} = \mathop {\hbox{min} }\limits_{i} a_{1ij} \,\left( {\text{cost criteria}} \right) \hfill \\ \end{array}} \right\}$$

*Step 4* Calculate the weighted normalized fuzzy decision matrix $\tilde{v}_{ij}$ as given below:

19 $$\tilde{v}_{ij} = \tilde{r}_{ij} \otimes \tilde{w}_{j} ,\quad {\text{i}} = 1,2, \ldots ,{\text{m}};\;{\text{j}} = 1,2, \ldots ,{\text{n}}$$

where $\tilde{w}_{j}$ is the weight of criterion c_j_.

*Step 5* Determine the fuzzy positive ideal solution (FPIS, A^+^) and fuzzy negative ideal solution (FNIS, A^−^) using the weighted normalized fuzzy decision matrix $\tilde{v}_{ij}$, as follows:

20 $$A^{ + } = \left( {\tilde{v}_{1}^{ + } ,\tilde{v}_{2}^{ + } , \ldots ,\tilde{v}_{n}^{ + } } \right)$$

21 $$A^{ - } = \left( {\tilde{v}_{1}^{ - } ,\tilde{v}_{2}^{ - } , \ldots ,\tilde{v}_{n}^{ - } } \right)$$

where $$\tilde{v}_{j}^{ + } = \mathop {\hbox{max} }\limits_{i} \left\{ {v_{ij} } \right\}$$ and $$\tilde{v}_{j}^{ - } = \mathop {\hbox{min} }\limits_{i} \left\{ {v_{ij} } \right\} .$$

*Step 6* Calculate the Euclidean distance ($D_{i}^{ + }$, $D_{i}^{ - }$) for each alternative ‘i’ from respectively $\tilde{v}_{j}^{ + }$ and $\tilde{v}_{j}^{ - }$ as follows.

22 $$D_{i}^{ + } = \sum\limits_{j = 1}^{n} {d_{v} \left( {\tilde{v}_{ij} ,\tilde{v}_{j}^{ + } } \right)}$$

23 $$D_{i}^{ - } = \sum\limits_{j = 1}^{n} {d_{v} \left( {\tilde{v}_{ij} ,\tilde{v}_{j}^{ - } } \right)}$$

*Step 7* Calculate the relative closeness coefficient (CC_i_) to the ideal solution of each alternative as follows:

24 $$CC_{i} = \, D_{i}^{ - } /\left( {D_{i}^{ + } + \, D_{i}^{ - } } \right)$$

*Step 8* Rank alternatives in decreasing order according to the closeness coefficient CC_i_, the most appropriate alternative should have the shortest distance from the fuzzy positive ideal solution and the farthest distance from the fuzzy negative ideal solution.

### Proposed hybrid fuzzy AHP–TOPSIS framework to evaluate and rank the GSCM practices

Several multi-criteria decision-making methods have been proposed in order to help decision makers to deal with complex situations by taking the right decision choice. Indeed, in this proposed framework, the FAHP method has been chosen thanks to its ability to structure and decompose a fuzzy decision-making problem into sub problems, then determine the weight of each element to classify it according to its relative importance. Concerning the process of ranking alternatives, we have chosen the fuzzy TOPSIS method due to its capability to deal with group decision making problems in uncertain environments. The decision group members can aid the implementation of the FAHP and fuzzy TOPSIS models by choosing linguistic terms that are ideal for GSCM practices evaluation and weighting the criteria as well as parameterizing the triangular fuzzy numbers corresponding to each linguistic term.

The main advantage of the proposed approach can be illustrated in terms of the evaluation of the identified alternatives and criteria. Indeed, the criteria evaluation process (FAHP process) is completely separated from that of the alternatives (fuzzy TOPSIS). This increases the efficiency and credibility of the final results compared to several other studies of which the assessment of alternatives and criteria is performed by the same analytical process. Another advantage is about the compensatory property of fuzzy TOPSIS process, in which the decision is based on the assumption that a bad performance of a GSCM practice on a particular criterion can be partially compensated by high ratings on other criteria, and its overall evaluation of performance and its rank will reflect that.

**Fig. 2:**
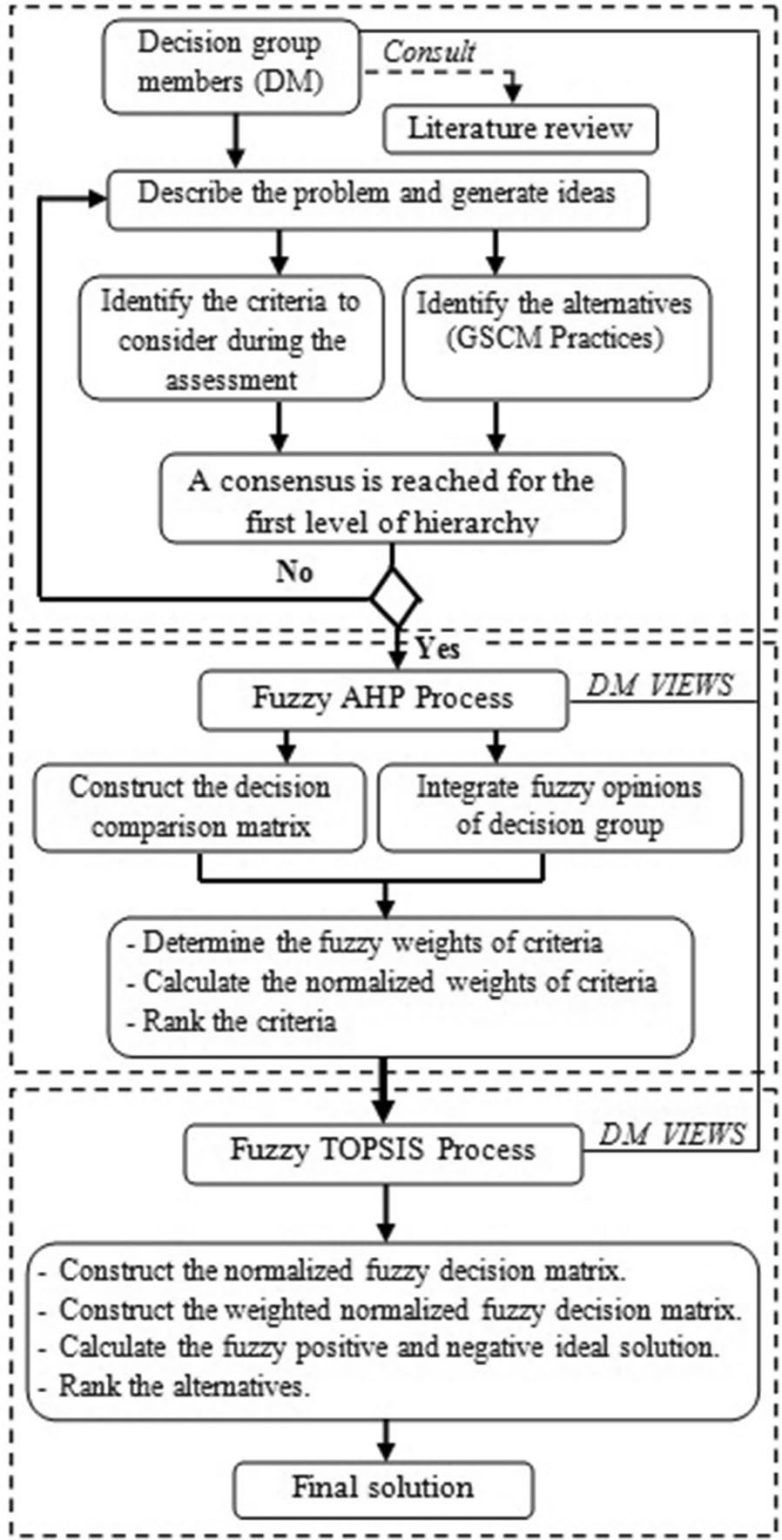
Proposed hybrid fuzzy AHP-TOPSIS framework to evaluate and rank GSCM Practices

The three major processes used in the proposed approach are explained in Fig. 2 as follows:

*Process I* This process occurs when three decision group members are formed in order to define objectives and specify all evaluation criteria needed to determine their favorable choice. All selected criteria and alternatives are determined through the literature review and validated by the decision group members.

*Process II* The FAHP process is used to decompose the decision-making problem into its constituent parts and construct hierarchies of the influential criteria, and then proceed to construct the fuzzy pairwise comparison matrices by integrating fuzzy opinions of decision group members on the relative importance of paired elements. Finally, FAHP process also calculates the fuzzy weight and final normalized weight of each criterion.

*Process III* The objective of this process is to evaluate and rank various GSCM practices considered by the decision group and the literature review. The importance weights of criteria obtained from the FAHP process are then considered as inputs to calculate the weighted normalized fuzzy decision matrix in the fuzzy TOPSIS process, which will allow us to determine the fuzzy positive and negative ideal solution, and then, identify the candidate alternative of the final ranking according to the closeness coefficient CC_i_.

## Numerical illustration

### Problem description

A Moroccan corporation of chemical industry established in Safi region is composed of a production chain which annually produces thousands of tons of phosphoric and sulfuric acid and several types of fertilizers. It contains sulfuric and phosphoric acid production plants, a gigantic workshop for repairing machines and spare parts as well as a production plant for electricity. This organization is interested in identifying, evaluating and ranking the GSCM best practices to improve its new manufacturing strategy towards sustainable development. In fact, the objective of this contribution is not to discover which GSCM practice is more important to adopt, but the ranking process using fuzzy AHP-TOPSIS methodology, allows this objective to be more comprehensive and systematic.

#### Identification of criteria

Each organization may use standardized criteria or any evaluation criteria arising from the organization’s core processes requirements. In this contribution, we present a set of selection criteria identified through the academic literature survey and proved by decision group members when evaluating GSCM practices. The objective is to select the most relevant criteria that have a direct or indirect association with GSCM in order to consider them in the evaluation process. Thus, three main criteria and ten sub-criteria to take into account are presented as follows:

Economic criteria (EC):

- EC1: Increase in productivity (Green et al. 2012).
- EC2: Decrease costs of material purchasing and energy consumption (De Giovanni and Vinzi 2012).
- EC3: Increased firm’s competitiveness (Lee et al. 2013).
- EC4: Increase in profitability (De Giovanni and Vinzi 2012).

Organizational criteria (OC):

- OC1: Lack of Human resources (Perron 2005).
- OC2: Lack of technological infrastructure and technical expertise (Perron 2005; Revell and Rutherfoord 2003).
- OC3: Lack of proper organizational structure to create and share knowledge (Ahmad and Daghfous 2010).

Environment criteria (EnC):

- EnC1: Improvement in environmental quality of products/processes (Zailani et al. 2012).
- EnC2: Reduction in air emissions, liquid and solid wastes (De Giovanni and Vinzi 2012).
- EnC3: Decrease in use of harmful/hazardous materials/components (De Giovanni and Vinzi 2012).

#### Identification of GSCM practices

In the following, we present some of the most selected GSCM practices. These practices (from Pr1 to Pr10) are proposed by several researches as explained below:

- Aligning GSCM improvements with the organization’s business goals for effective strategic value (Greenprof 2015).
- Optimizing the operations of both integrated logistics and corresponding used-product reverse logistics in a given green-supply chain (Sheu et al. 2005).
- Focusing on source reduction programs for more valuable improvements (Greenprof 2015).
- Complying with legal environmental requirements and auditing programs (Kannan et al. 2014).
- Considering the existing business model when planning GSCM projects (Greenprof 2015).
- Acquisition of the cleanest technologies by the company (Kannan et al. 2014).
- Using green supply chain analysis as a catalyst for innovation (Greenprof 2015).
- Evaluating the GSCM as a single life cycle system.
- Strengthening the cultural cohesions and co-operation in SC members (Wong and Wong 2011).
- Making strategic alliances among the supply chain partners for positive impact on SC performance (Wong and Wong 2011).

#### The proposed hierarchical structure

**Fig. 3:**
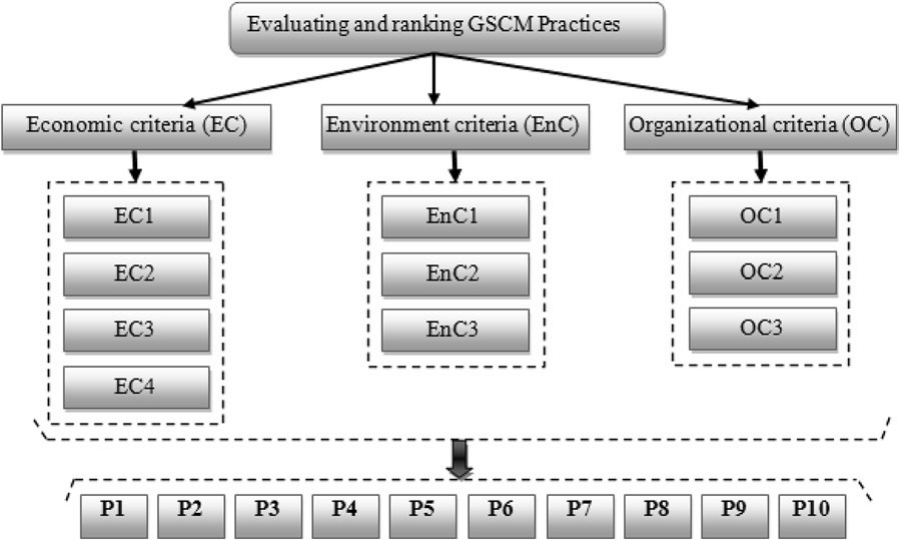
Hierarchical analysis structure to evaluate GSCM practices

As explained before, the aim of this contribution is to propose a hybrid fuzzy AHP-TOPSIS framework for evaluating and ranking GSCM practices provided through a literature review and decision group. The hierarchical structure used in this decision situation consists of four levels: as shown in Fig. 3. The objective is shown in the highest level and divided into three main criteria on the second level, while ten sub-criteria are identified on the third level. The last level of hierarchy includes ten selected practices as explained below:

### Calculate weight of criteria using FAHP process

Evaluations of the weight of criteria are performed by the decision group according to the linguistic terms depicted in Table 2. Hence, the comparative judgments for the main criteria are provided by three decision group members as shown in Table 3, see also “Appendix 2”. Those judgments are aggregated using Eqs. (8–10) for triangular fuzzy numbers as illustrated in Table 4. The fuzzy weight is then obtained on the basis of the geometric average of TFNs using Eqs. (12, 13), and the final normalized weight is provided using Eqs. (14, 15) as shown in Table 5.

**Table 2:** Membership function of linguistic scale (Gumus 2009)

Linguistic variables	Fuzzy number	TFN scale
Very good (VG)	$\tilde{9}$	(7, 9, 9)
Good (Gd)	$\tilde{7}$	(5, 7, 9)
Preferable (P)	$\tilde{5}$	(3, 5, 7)
Weak advantage (WA)	$\tilde{3}$	(1, 3, 5)
Equal (EQ)	$\tilde{1}$	(1, 1, 1)
Less WA	$\tilde{3}^{ - 1}$	(1/5, 1/3, 1)
Less P	$\tilde{5}^{ - 1}$	(1/7, 1/5, 1/3)
Less G	$\tilde{7}^{ - 1}$	(1/9, 1/7, 1/5)
Less VG	$\tilde{9}^{ - 1}$	(1/9, 1/9, 1/7)

**Table 3:** Comparative judgments for the criteria weight made by decision makers using linguistic variables

Objective	EC			EnC			OC		
	M_1_	M_2_	M_3_	M_1_	M_2_	M_3_	M_1_	M_2_	M_3_
EC	EQ	EQ	EQ	L. WA	WA	P	VG	P	P
EnC	WA	L. WA	L. P	EQ	EQ	EQ	P	VG	G
OC	L. VG	L. P	L. P	L. P	L. VG	L. G	EQ	EQ	EQ

**Table 4:** Triangular fuzzy numbers of the aggregated judgments for the criteria weight

Objective	EC	EnC	OC
EC	(1.000, 1.000, 1.000)	(0.143, 0.585, 5.000)	(3.000, 6.082, 9.000)
EnC	(0.200, 1.442, 5.000)	(1.000, 1.000, 1.000)	(3.000, 6.804, 9.000)
OC	(0.111, 0.164, 0.333)	(0.111, 0.147, 0.333)	(1.000, 1.000, 1.000)

**Table 5:** The geometric average ($\tilde{Z}_{i}$), fuzzy weight ($\tilde{W}_{i}$) and final normalized weight (*NW*_*i*_)

Main criteria	Geometric average ($\tilde{Z}_{i}$)	Fuzzy weight ($\tilde{W}_{i}$)	Defuzification (*W*_*i*_)	Final normalized weight (*NW*_*i*_)
EC	(0.754, 1.526, 3.557)	(0.412, 0.386, 0.468)	0.422	0.422
EnC	(0. 843, 2.141, 3.557)	(0.461, 0.541, 0.468)	0.490	0.490
OC	(0.231, 0.289, 0.481)	(0.126, 0.073, 0.063)	0.088	0.088

Following the same computational steps (Tables 3, 4, 5), we get the final evaluation results as shown in Table 6, including the final weight of each criterion and sub criterion.

**Table 6:** Final evaluation results of criteria weight

Main criteria	Weight of main criteria	Evaluation criteria	Hierarchy fuzzy weight	Total fuzzy weight/normalized weight	Ranking importance
EC	(0.412, 0.386, 0.468)	EC1	(0.199, 0.174, 0.161)	(0.082, 0.067, 0.075) 0.075	5
		EC2	(0.114, 0.140, 0.146)	(0.047, 0.054, 0.068) 0.056	7
		EC3	(0.138, 0.116, 0.161)	(0.057, 0.045, 0.075) 0.059	4
		EC4	(0.549, 0.570, 0.532)	(0.226, 0.220, 0.249) 0.232	2
EnC	(0.461, 0.541, 0.468)	EnC1	(0.600, 0.633, 0.560)	(0.277, 0.342, 0.262) 0.294	1
		EnC2	(0.268, 0.260, 0.319)	(0.124, 0.141, 0.149) 0.138	3
		EnC3	(0.132, 0.106, 0.121)	(0.061, 0.057, 0.057) 0.058	6
OC	(0.126, 0.073, 0.063)	OC1	(0.132, 0.106, 0.121)	(0.017, 0.008, 0.008) 0.011	10
		OC2	(0.268, 0.260, 0.319)	(0.034, 0.019, 0.020) 0.024	9
		OC3	(0.600, 0.633, 0.560)	(0.076, 0.046, 0.035) 0.052	8

### Fuzzy TOPSIS process

During fuzzy TOPSIS process, the importance weights assigned to the selected criteria using FAHP will be used as input to evaluate and rank alternatives.

The computational procedure to follow during this proposed process is summarized as explained below:

*Step 1* The rating of alternatives with respect to each criterion is performed by three members of the decision-making group (see the additional file provided with the paper for fuzzy TOPSIS process) using linguistic rating variables with (TFN) numbers shown in Table 7.

**Table 7:** Transformation for fuzzy membership functions

Linguistic expression	Triangular fuzzy numbers
Very important (VP)	(0.75, 0.90, 1.00)
Important (P)	(0.55, 0.70, 0.85)
Medium importance (MP)	(0.35, 0.50, 0.65)
Insufficient (I)	(0.15, 0.30, 0.45)
Very insufficient (VI)	(0.00, 0.10, 0.25)

*Step 2* The fuzzy decision matrix is constructed by aggregating the fuzzy rating $\tilde{g}_{ij}$ of alternative A_i_ under criterion C_j_ as explained in Table 8.

**Table 8:** The fuzzy decision matrix resulting from aggregation of the judgments

	OC1	OC2	OC3	EC1	EC2	EC3	EC4	EnC1	EnC2	EnC3
P 1	(0.150, 0.500, 0.850)	(0.150, 0.500, 0.850)	(0.150, 0.500, 0.850)	(0.350, 0.633, 1.000)	(0.150, 0.367, 0.650)	(0.350, 0.567, 0.850)	(0.150, 0.367, 0.650)	(0.350, 0.633, 0.850)	(0.150, 0.433, 0.650)	(0.150, 0.567, 0.850)
P 2	(0.350, 0.500, 0.650)	(0.350, 0.633, 1.000)	(0.350, 0.567, 0.850)	(0.150, 0.433, 0.650)	(0.150, 0.500, 0.850)	(0.150 0.633 1.000,)	(0.350, 0.633, 1.000)	(0.150, 0.500, 0.850)	(0.350, 0.567, 0.850)	(0.350, 0.567, 0.850)
P 3	(0.000, 0.367, 0.850)	(0.150, 0.500, 0.850)	(0.150, 0.433, 0.850)	(0.550, 0.700, 0.850)	(0.150, 0.300, 0.450)	(0.000, 0.300 0.650,)	(0.150, 0.500, 0.850)	(0.000, 0.367, 0.850)	(0.150, 0.567, 0.850)	(0.000, 0.500, 0.850)
P 4	(0.000, 0.367, 0.650)	(0.150, 0.433, 0.650)	(0.350, 0.633, 0.850)	(0.150, 0.500, 0.850)	(0.350, 0.633, 0.850)	(0.350, 0.633, 0.850)	(0.150, 0.367, 0.650)	(0.350, 0.633, 0.850)	(0.000, 0.367, 0.650)	(0.000, 0.367, 0.850)
P 5	(0.350, 0.633, 0.850)	(0.000, 0.233, 0.450)	(0.000, 0.433, 0.850)	(0.150, 0.433, 0.850)	(0.150, 0.500, 0.850)	(0.150, 0.433, 0.650)	(0.000, 0.367, 0.850)	(0.000, 0.300, 0.650)	(0.150, 0.433, 0.850)	(0.150, 0.500, 0.850)
P 6	(0.350, 0.633, 0.850)	(0.150, 0.567, 1.000)	(0.150, 0.367, 0.650)	(0.150, 0.500, 0.850)	(0.350, 0.567, 0.850)	(0.550, 0.767, 1.000)	(0.350, 0.633, 1.000)	(0.150, 0.567, 0.850)	(0.350, 0.633, 0.850)	(0.350, 0.567, 0.850)
P 7	(0.150, 0.500, 0.850)	(0.000, 0.500, 0.850)	(0.150, 0.433, 0.650)	(0.150, 0.633, 1.000)	(0.150, 0.433, 0.850)	(0.150, 0.567, 0.850)	(0.000, 0.567, 1.000)	(0.350, 0.633, 0.850)	(0.150, 0.500, 0.850)	(0.150, 0.567, 0.850)
P 8	(0.150, 0.567, 0.850)	(0.150, 0.433, 0.650)	(0.000, 0.367, 0.850)	(0.150, 0.433, 0.650)	(0.150, 0.500, 0.850)	(0.150, 0.500, 0.850)	(0.150, 0.367, 0.650)	(0.000, 0.367, 0.850)	(0.150, 0.433, 0.850)	(0.150, 0.567, 0.850)
P 9	(0.000, 0.367, 0.850)	(0.350, 0.567, 0.850)	(0.150, 0.567, 0.850)	(0.150, 0.633, 1.000)	(0.150, 0.367, 0.650)	(0.000, 0.300, 0.650)	(0.350, 0.633, 1.000)	(0.000, 0.500, 0.850)	(0.150, 0.567, 0.850)	(0.000, 0.367, 0.850)
P 10	(0.150, 0.367, 0.650)	(0.000, 0.233, 0.450)	(0.150, 0.500, 0.850)	(0.150, 0.500, 0.850)	(0.150, 0.500, 0.850)	(0.150, 0.567, 0.850)	(0.000, 0.300, 0.650)	(0.150, 0.433, 0.850)	(0.150, 0.433, 0.850)	(0.150, 0.500, 0.850)

*Step 3* The normalized fuzzy decision matrix is constructed using Eqs. (17, 18), as mentioned in Table 9.

**Table 9:** Normalized fuzzy decision matrix (*r*_*ij*_)

	OC1	OC2	OC3	EC1	EC2	EC3	EC4	EnC1	EnC2	EnC3
P 1	(0.176, 0.588, 1.000)	(0.150, 0.500, 0.850)	(0.176, 0.588, 1.000)	(0.350, 0.633, 1.000)	(0.176, 0.431, 0.765)	(0.350, 0.567, 0.850)	(0.150, 0.367, 0.650)	(0.412, 0.745, 1.000)	(0.176, 0.510, 0.765)	(0.176, 0.667, 1.000)
P 2	(0.412, 0.588, 0.765)	(0.350, 0.633, 1.000)	(0.412, 0.667, 1.000)	(0.150, 0.433, 0.650)	(0.176, 0.588, 1.000)	(0.150, 0.633, 1.000)	(0.350, 0.633, 1.000)	(0.176, 0.588, 1.000)	(0.412, 0.667, 1.000)	(0.412, 0.667, 1.000)
P 3	(0.000, 0.431, 1.000)	(0.150, 0.500, 0.850)	(0.176, 0.510, 1.000)	(0.550, 0.700, 0.850)	(0.176, 0.353, 0.529)	(0.000, 0.300, 0.650)	(0.150, 0.500, 0.850)	(0.000, 0.431, 1.000)	(0.176, 0.667, 1.000)	(0.000, 0.588, 1.000)
P 4	(0.000, 0.431, 0.765)	(0.150, 0.433, 0.650)	(0.412, 0.745, 1.000)	(0.150, 0.500, 0.850)	(0.412, 0.745, 1.000)	(0.350, 0.633, 0.850)	(0.150, 0.367, 0.650)	(0.412, 0.745, 1.000)	(0.000, 0.431, 0.765)	(0.000, 0.431, 1.000)
P 5	(0.412, 0.745, 1.000)	(0.000, 0.233, 0.450)	(0.000, 0.510, 1.000)	(0.150, 0.433, 0.850)	(0.176, 0.588, 1.000)	(0.150, 0.433, 0.650)	(0.000, 0.367, 0.850)	(0.000, 0.353, 0.765)	(0.176, 0.510, 1.000)	(0.176, 0.588, 1.000)
P 6	(0.412, 0.745, 1.000)	(0.150, 0.567, 1.000)	(0.176, 0.431, 0.765)	(0.150, 0.500, 0.850)	(0.412, 0.667, 1.000)	(0.550, 0.767, 1.000)	(0.350, 0.633, 1.000)	(0.176, 0.667, 1.000)	(0.412, 0.745, 1.000)	(0.412, 0.667, 1.000)
P 7	(0.176, 0.588, 1.000)	(0.000, 0.500, 0.850)	(0.176, 0.510, 0.765)	(0.150, 0.633, 1.000)	(0.176, 0.510, 1.000)	(0.150, 0.567, 0.850)	(0.000, 0.567, 1.000)	(0.412, 0.745, 1.000)	(0.176, 0.588, 1.000)	(0.176, 0.667, 1.000)
P 8	(0.176, 0.667, 1.000)	(0.150, 0.433, 0.650)	(0.000, 0.431, 1.000)	(0.150, 0.433, 0.650)	(0.176, 0.588, 1.000)	(0.150, 0.500, 0.850)	(0.150, 0.367, 0.650)	(0.000, 0.431, 1.000)	(0.176, 0.510, 1.000)	(0.176, 0.667, 1.000)
P 9	(0.000, 0.431, 1.000)	(0.350, 0.567, 0.850)	(0.176, 0.667, 1.000)	(0.150, 0.633, 1.000)	(0.176, 0.431, 0.765)	(0.000, 0.300, 0.650)	(0.350, 0.633, 1.000)	(0.000, 0.588, 1.000)	(0.176, 0.667, 1.000)	(0.000, 0.431, 1.000)
P 10	(0.176, 0.431, 0.765)	(0.000, 0.233, 0.450)	(0.176, 0.588, 1.000)	(0.150, 0.500, 0.850)	(0.176, 0.588, 1.000)	(0.150, 0.567, 0.850)	(0.000, 0.300, 0.650)	(0.176, 0.510, 1.000)	(0.176, 0.510, 1.000)	(0.176, 0.588, 1.000)

*Step 4* The weighted normalized fuzzy decision matrix is obtained by applying Eq. (19) as in Table 10 using the fuzzy weights of the criteria already calculated from fuzzy AHP process.

**Table 10:** Weighted normalized fuzzy decision matrix (*v*_*ij*_)

	*OC1*	*OC2*	*OC3*	*EC1*	*EC2*	*EC3*	*EC4*	*EnC1*	*EnC2*	*EnC3*
Weights of criteria	(0.017, 0.008, 0.008)	(0.034, 0.019, 0.020)	(0.076, 0.046, 0.035)	(0.082, 0.067, 0.075)	(0.047, 0.054, 0.068)	(0.057, 0.045, 0.075)	(0.226, 0.220, 0.249)	(0.277, 0.342, 0.262)	(0.124, 0.141, 0.149)	(0.061, 0.057, 0.057)
P 1	(0.003, 0.005, 0.008)	(0.005, 0.009, 0.017)	(0.013, 0.027, 0.035)	(0.029, 0.043, 0.075)	(0.008, 0.023, 0.052)	(0.020, 0.025, 0.064)	(0.034, 0.081, 0.162)	(0.114, 0.255, 0.262)	(0.022, 0.072, 0.114)	(0.011, 0.038, 0.057)
P 2	(0.007, 0.005, 0.006)	(0.012, 0.012, 0.020)	(0.031, 0.031, 0.035)	(0.012, 0.029, 0.049)	(0.008, 0.032, 0.068)	(0.009, 0.028, 0.075)	(0.079, 0.139, 0.249)	(0.049, 0.201, 0.262)	(0.051, 0.094, 0.149)	(0.025, 0.038, 0.057)
P 3	(0.000, 0.003, 0.008)	(0.005, 0.009, 0.017)	(0.013, 0.024, 0.035)	(0.045, 0.047, 0.064)	(0.008, 0.019, 0.036)	(0.000, 0.013, 0.049)	(0.034, 0.110, 0.212)	(0.000, 0.148, 0.262)	(0.022, 0.094, 0.149)	(0.000, 0.034, 0.057)
P 4	(0.000, 0.003, 0.006)	(0.005, 0.008, 0.013)	(0.031, 0.034, 0.035)	(0.012, 0.034, 0.064)	(0.019, 0.040, 0.068)	(0.020, 0.028, 0.064)	(0.034, 0.081, 0.162)	(0.114, 0.255, 0.262)	(0.000, 0.061, 0.114)	(0.000, 0.025, 0.057)
P 5	(0.007, 0.006, 0.008)	(0.000, 0.004, 0.009)	(0.000, 0.024, 0.035)	(0.012, 0.029, 0.064)	(0.008, 0.032, 0.068)	(0.009, 0.019, 0.049)	(0.000, 0.081, 0.212)	(0.000, 0.121, 0.200)	(0.022, 0.072, 0.149)	(0.011, 0.034, 0.057)
P 6	(0.007, 0.006, 0.008)	(0.005, 0.011, 0.020)	(0.013, 0.020, 0.027)	(0.012, 0.034, 0.064)	(0.019, 0.036, 0.068)	(0.031, 0.034, 0.075)	(0.079, 0.139, 0.249)	(0.049, 0.228, 0.262)	(0.051, 0.105, 0.149)	(0.025, 0.038, 0.057)
P 7	(0.003, 0.005, 0.008)	(0.000, 0.009, 0.017)	(0.013, 0.024, 0.027)	(0.012, 0.043, 0.075)	(0.008, 0.028, 0.068)	(0.009, 0.025, 0.064)	(0.000, 0.125, 0.249)	(0.114, 0.255, 0.262)	(0.022, 0.083, 0.149)	(0.011, 0.038, 0.057)
P 8	(0.003, 0.005, 0.008)	(0.005, 0.008, 0.013)	(0.000, 0.020, 0.035)	(0.012, 0.029, 0.049)	(0.008, 0.032, 0.068)	(0.009, 0.022, 0.064)	(0.034, 0.081, 0.162)	(0.000, 0.148, 0.262)	(0.022, 0.072, 0.149)	(0.011, 0.038, 0.057)
P 9	(0.000, 0.003, 0.008)	(0.012, 0.011, 0.017)	(0.013, 0.031, 0.035)	(0.012, 0.043, 0.075)	(0.008, 0.023, 0.052)	(0.000, 0.013, 0.049)	(0.079, 0.139, 0.249)	(0.000, 0.201, 0.262)	(0.022, 0.094, 0.149)	(0.000, 0.025, 0.057)
P 10	(0.003, 0.003, 0.006)	(0.000, 0.004, 0.009)	(0.013, 0.027, 0.035)	(0.012, 0.034, 0.064)	(0.008, 0.032, 0.068)	(0.009, 0.025, 0.064)	(0.000, 0.066, 0.162)	(0.049, 0.175, 0.262)	(0.022, 0.072, 0.149)	(0.011, 0.034, 0.057)

*Step 5* The fuzzy positive ideal solution FPIS and fuzzy negative ideal solution FNIS are performed using Eqs. (20, 21) as in Table 11, and the distance of each alternative from FPIS and FNIS is calculated using Eqs. (22, 23) as shown in Table 12.

**Table 11:** Fuzzy positive and negative ideal solution

A^+^	(0.007, 0.006, 0.008)	(0.012, 0.012, 0.020)	(0.031, 0.034, 0.035)	(0.045, 0.047, 0.075)	(0.019, 0.040, 0.068)	(0.031, 0.034, 0.075)	(0.079, 0.139, 0.249)	(0.114, 0.255, 0.262)	(0.051, 0.105, 0.149)	(0.025, 0.038, 0.057)
A^−^	(0.000, 0.003, 0.006)	(0.000, 0.004, 0.009)	(0.000, 0.020, 0.027)	(0.012, 0.029, 0.049)	(0.008, 0.019, 0.036)	(0.000, 0.013, 0.049)	(0.000, 0.066, 0.162)	(0.000, 0.121, 0.200)	(0.000, 0.061, 0.114)	(0.000, 0.025, 0.057)

**Table 12:** The related closeness coefficients (*CC*_*i*_) and final ranking of GSCM practices

Alternatives	Distance D_*i*_^+^	Distance *D*_*i*_^−^	CC_i_	Rank
P 1	0.160	0.217	0.575	4
P 2	0.106	0.274	0.720	2
P 3	0.238	0.158	0.398	7
P 4	0.168	0.203	0.547	5
P 5	0.285	0.107	0.273	10
P 6	0.083	0.295	0.781	1
P 7	0.143	0.269	0.654	3
P 8	0.269	0.137	0.338	9
P 9	0.184	0.217	0.541	6
P 10	0.243	0.141	0.366	8

**Fig. 4:**
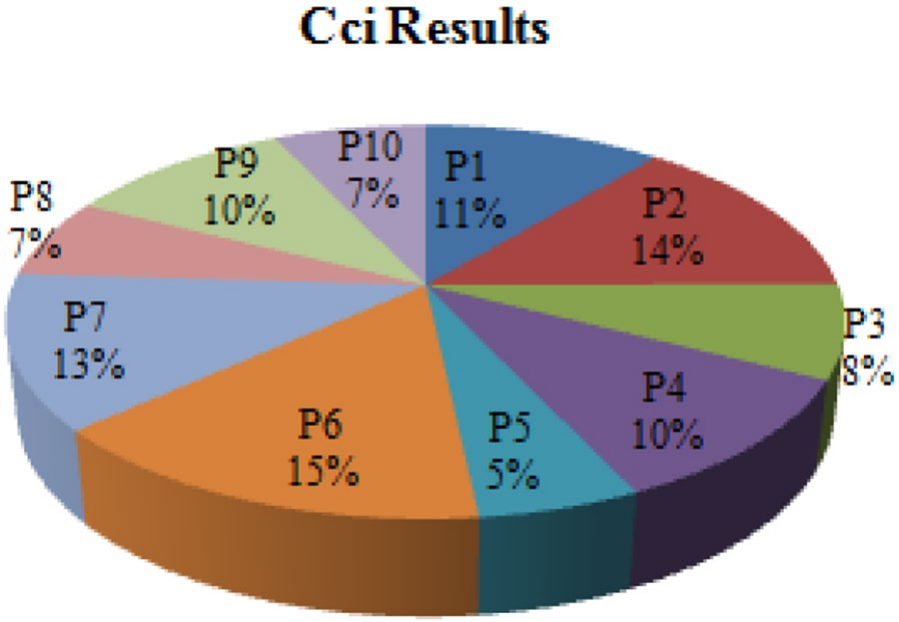
Final results

*Step 6* The closeness coefficient of each alternative is determined by applying Eq. (24), and the final ranking of the alternatives (GSCM practices) depending on the descending order of closeness coefficient is provided as shown in Table 12 and Fig. 4.

### Simulation results and sensitivity analysis

As shown in Table 6, the final results of analysis during the first process show that environmental criteria remain the most important ones compared to other main criteria. This explains that the decision-making group gives more attention to environmental impacts, strengthened through the sub-criterion ‘EnC1’ with an important weight of 0.294, followed by the economic sub-criterion ‘EC4’ with an important weight of 0.232. The low importance is given to the organizational criteria due to the nature of our case study which is more focused on sustainability.

As also illustrated in Fig. 4, the final evaluation results of effective GSCM practices are provided. In fact, the relative score of each alternative is displayed on the basis of the contribution of each of the evaluation criteria. The most appropriate practices are those with the highest score according to the final ranking presented in Table 12, which revealed that the preferred GSCM practices, according to decision makers and experts, were P6 with an adoption percentage of 15 %, followed by P2 (14 %) and P7 (13 %) until the last preferred practice P5 (5 %).

In order to show the robustness of the proposed methodology and measure the influence of decision makers’ risks to the final ranking, a sensitivity analysis is performed which is illustrated in “Appendix 1”. To this end, the most important criterion’s weight (0.294 as mentioned in Table 6) is gradually exchanged with another criterion’s weight, while keeping all the other weights the same, and the influence on the final decisions is investigated. This operation is carried out respectively for each criterion and the results are properly detailed in “Appendix 1”.

The intended objective, as suggested in several contributions as in Mousavi et al. (2013), Zhu et al. (2015) and Mosadeghi et al. (2015), is to check for the feasible changes that may affect the final rankings provided in Table 12. Therefore, ten combinations declared as conditions are investigated. The original result mentioned in Table 12 is described as the main condition. The comparisons show that P6 remains the most preferred choice in seven conditions out of nine. P2 is ranked as the second best practice in seven conditions compared to other alternatives. Also, P7 is ranked as the third best practice in six conditions and so on.

The final results of the sensitivity analysis demonstrate that the alternatives’ ranking has changed significantly according to equal weights of the criteria, which explains that the criteria weights found consistently form a significant step in the proposed integrated approach. In addition to the identification of criteria devoted to the specific GSCM, conclusions and remarks can be derived with respect to the importance of criteria. In order to generate comprehensive insights, we reflect on higher level criteria, which are presented in the problem description section. Consequently, the conducted sensitivity analysis shows that the weights have impacts on the ranking of alternatives. This will allow decision makers to improve their decision-making process by adjusting weighting, scoring and performing sensitivity analyses.

With regard to future research we recommend to investigate the general applicability of the developed approach for the evaluation of GSCM practices in manufacturing companies for different environmental competitive strategies, and more business opportunities. In this concern, we are currently working on the development of multi-criteria analysis prototype based on the FAHP and fuzzy TOPSIS algorithms that explicitly include the evaluation of GSCM practices in this prototype, and especially, the identified criteria performed on the basis of the literature review to evaluate those practices. Moreover, several applicative studies could be conducted in order to evaluate the usability of a tool such as this for supporting the evaluation of GSCM practices. However, to conduct this line of research, the proposed model should be implemented as an expert system.

## Conclusion

The present study explores the use of fuzzy AHP-TOPSIS framework to allow decision makers to identify, evaluate and rank the effective GSCM practices in order to implement them in the production process of their corporations. In fact, the successful implementation of these practices requires raising the profile of environmental projects by articulating project value in terms of business value, and creating the project to work within the organizational culture. The methodology we adopt in this contribution involves the use of triangular fuzzy numbers to evaluate the fuzzy opinions of decision group members, and consists of three major processes. The first is to define objectives and specify all evaluation criteria needed to take into consideration when evaluating GSCM practices. The necessary criteria for performance rankings of GSCM are determined from the literature review and validated by three decision group members. Secondly, FAHP process is used to structure the problem and determine the importance weight of the selected criteria, and finally, fuzzy TOPSIS process uses these weights as inputs to generate an overall performance score by evaluating and measuring the performance of each GSCM practice.

For further studies, different multi-criteria decision making methods such as fuzzy PROMETHEE, VIKOR and ELECTRE can be used as mentioned in the literature, and the comparison of the results can be presented. The intended objective is to support organizations in their decisions about their priorities of implementing GSCM practices in order to assure future sustainable strategies. In this context, a sensitivity analysis is performed for the case study to contribute to better assessing the risk of decision makers’ perception. We ultimately believe that the results provided are more objective and the vagueness is quantified and addressed properly.
